# Soil N and P nutrient metabolism affected by fungal community in larch plantation

**DOI:** 10.3389/fmicb.2026.1658803

**Published:** 2026-03-23

**Authors:** Xingran Mo, Yang Liu, Zhongliang Huang, Huizhen Yu, Jing Huang, Zijian Wu, Mengjiao Tan, Huixin Fan, Fengfeng Ma, Baiquan Zeng

**Affiliations:** 1 College of Life and Environmental Sciences, Central South University of Forestry and Technology, Changsha, Hunan, China; 2 State Key Laboratory of Utilization of Woody Oil Resource, Hunan Academy of Forestry, Changsha, Hunan, China; 3 College of Resources and Environment, Yangtze University, Wuhan, Hubei, China; 4 Faculty of Arts and Social Sciences, The University of Sydney, Sydney, NSW, Australia; 5 Yuelushan Laboratory, Changsha, Hunan, China

**Keywords:** forest management, *Larix kaempferi*, rhizosphere microbiome, soil nitrogen, soil phosphate, tree stand age

## Abstract

**Introduction:**

The time-response mechanism of soil nitrogen (N) and phosphorus (P) nutrients across different stand ages remains intricate and inadequately quantified, particularly unclear is the effects of rhizosphere soil microbial communities, which serve as crucial drivers on soil N and P nutrients. This study delved into the effects of soil fungal community on the shifts of soil physicochemical properties and their correlations between N-P distribution within rhizosphere of *Larix kaempferi* (Japanese larch) with different tree stands.

**Methods:**

This study investigated the responses of soil nitrogen (N) and phosphorus (P) along a stand age gradient (young: <20 years; mid-aged: 20–30 years; near-mature: 30–40 years; mature: >40 years) in *Larix kaempferi* forests, with a focus on the associations between rhizosphere microbial communities and soil nutrient dynamics. By covering key developmental stages of forest succession, we examined age-related changes in rhizosphere soil N and P concentrations, soil physicochemical properties, and fungal community structure.

**Results:**

The results showed that fungal community structure gradually diversified from young to near-mature forests and became more stable in the mature forest stage. Differences in forest age were associated with changes in the availability and distribution of soil N and P nutrients, accompanied by shifts in the relative abundance of microbial functional genes related to N and P cycling. In particular, the abundance of P cycling–related functional genes showed patterns consistent with soil N and P variations, while N fixation–related functional genes exhibited the highest abundance in the middle-aged forest stage.

**Discussion:**

Overall, variations in stand development along the forest age gradient were closely linked to changes in soil nutrient distribution and rhizosphere microbial biomass, highlighting the potential role of rhizosphere microbial communities in soil N and P cycling in larch plantation ecosystems.

## Graphical abstract

**Figure:**
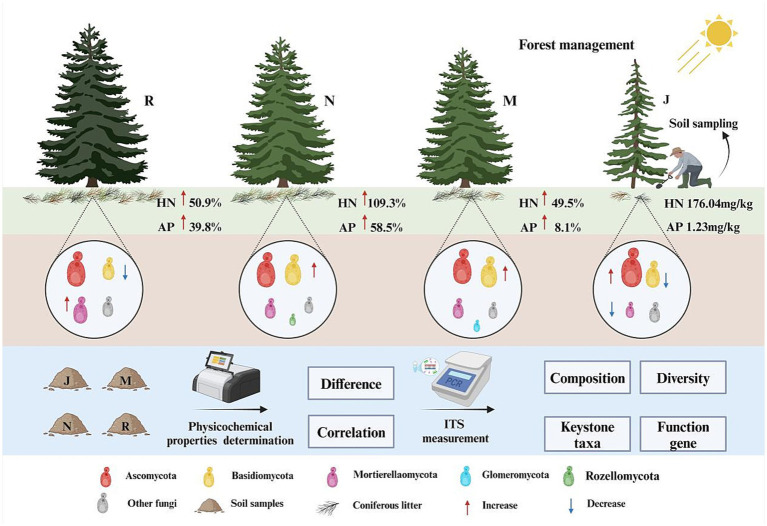

## Highlights

- Fungi was dominated by Ascomycota, Basidiomycota, and Mortierellomycota.
- Soil nutrients are the key factors affecting the change of fungal community.
- TP and AP were significantly positively correlated with HN.
- HN and AP increased 109.3, 58.5% in near-mature stand than young stand.
- PhnP, gcd, ppa, and ppx are the key genes driving to the increase of AP.

## Introduction

Soil nitrogen (N) and phosphorus (P), as essential nutrient elements limiting plant growth (Ågren et al., 2012; Du et al., 2021), serve as pivotal indicators of evaluating soil quality, environmental changes, and factors impeding the advancement of plantation forest productivity (Cunha et al., 2022). Plantation forests not only support the wood industry to promote local economic development, but also help alleviate global climate challenges (Bukoski et al., 2022). As forest stands age, the demand for N and P elements by understory vegetation and soil microorganisms undergoes dynamic shifts (Zhang et al., 2023). Hence, comprehending the dynamics of soil N and P during forest age transitions is instrumental in making informed management decisions concerning these nutrients in forests, thereby enhancing plantation productivity.

The management of plantation forests often encounters numerous challenges (Barrette et al., 2023; Jones et al., 2023). Forest age stands out as a pivotal factor in artificial afforestation, exerting influence on the distribution pattern of soil nutrients through alterations in the structure and material composition of forest stands (Fang et al., 2023; Li X. et al., 2023). Forest productivity, represented by tree biomass, peaks in the early stages of stand time series (young forest stage), with trees allocating more biomass to stems, leaves and branches to stimulate growth, nutrient element absorption also intensifies during the initial stages of the time series. Notably, forest age may significantly influence litter yield and quality (Ge et al., 2013). Within forest ecosystems, a substantial amount of N is sequestered in forest biomass via absorption and transformation before being deposited into the soil as litter (Cheng et al., 2019). Consequently, the demand for trees directly impacts the dynamic fluctuations of soil N (Gao et al., 2023). Studies have identified a prevalent phenomenon of P deficiency in subtropical forest ecosystems (Cui et al., 2022), as the P cycle predominantly relies on internal circulation within the system (Lang et al., 2016). Thus, the distribution and cycling patterns of soil P in forest ecosystems are shaped by the P requirements of trees (Wu et al., 2020).

The decomposition and transformation utilization of litter by microorganisms constitute a crucial aspect of driving nutrient cycling, including N and P (Ball et al., 2014; Pang et al., 2022). Microbial regulation of soil nutrient cycling dynamically responds to nutrient inputs through changes in biomass and community structure (Ma et al., 2023). For instance, soil microorganisms can liberate organic P from mineralized soil by secreting hydrolytic enzymes like acid and alkaline phosphatase (Wan et al., 2022). Simultaneously, they can modulate the microbial community structure involved in P cycling, thus mitigating the constraint of available P (Honvault et al., 2021). Microbial biomass N represents the primary component of soil organic N, ranking second only to plant biomass N (Li et al., 2021). Their cells store a considerable amount of unstable N, serving as a N source and reservoir within forest ecosystems. Additionally, microbial communities partake in various N metabolism processes, converting complex organic N compounds in soil into smaller molecules, thereby facilitating the transformation and utilization by plants and microorganisms (Gu et al., 2023). Consequently, the ecological behavior of microbial communities emerges as a significant determinant influencing soil N and P cycling and the progression of artificial forest productivity (Liu R. et al., 2024).

In this study, larch plantations were selected for analysis to discern changes in rhizosphere soil N and P concentrations, physicochemical properties, and microbial community structure. The effects of soil fungi community were revealed on the shifts of soil physicochemical properties and their correlations between N-P distribution within rhizosphere of larch with different tree stands. This study aims to elucidate the turnover process of N and P elements in rhizosphere soil under diverse forest ages, aiding in the formulation of N and P management strategies for larch plantation forests. Additionally, it will provide a theoretical foundation for the sustainable development of plantation forests.

## Materials and methods

### Site description

**Figure 1:**
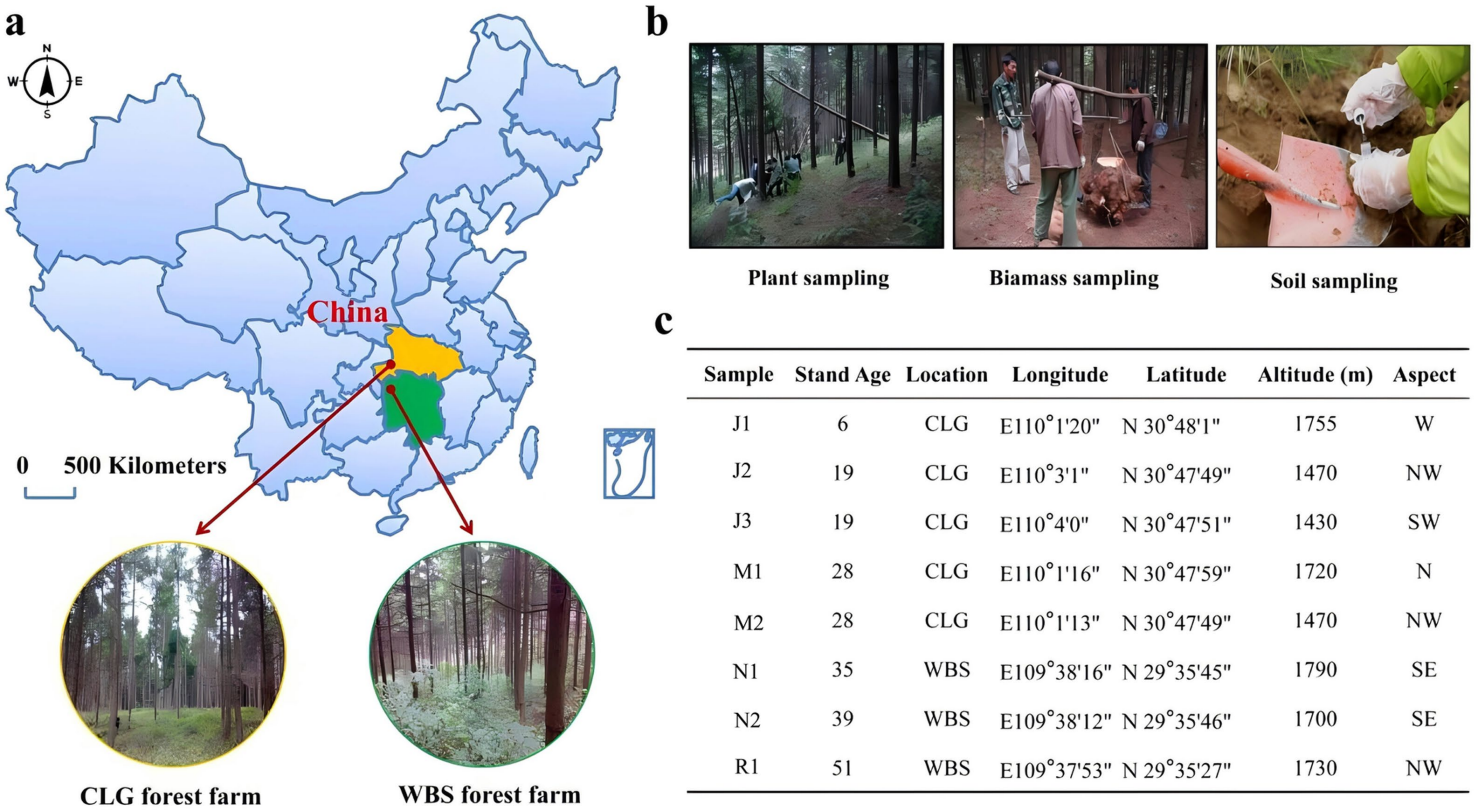
Sampling locations and methods. **(a)** A map showing the locations of the CLG and WBS forest farms in China. The arrows indicate the distances between the two sites, each located approximately 500 kilometers apart. **(b)** Field sampling activities: left image shows plant sampling, middle image shows biomass sampling, and right image depicts soil sampling at the study sites. **(c)** A table providing detailed information about the samples, including stand age, location, coordinates (longitude, latitude), altitude (m), and aspect of the sample sites. CLG forest farm (Jianshi County, Enshi, Hubei Province, China). WBS forest farm (Longshan County, Xiangxi, Hunan Province, China). Young stand (J1–J3); middle aged stand (M1, M2); near-mature stand (N1, N2); mature stand (R1). N, north; S, south; W, west; E, east.

Japanese larch, native to Honshu Island, Japan, is a medium to large deciduous coniferous tree renowned for its resilient and enduring wood, primarily utilized in construction purposes. Its global introduction to forestry plantations in European countries of the northern hemisphere occurred during the late 19th century. China embarked on the introduction of Japanese larch in the mid-20th century for productive afforestation to satisfy the growing wood demand. The sampling sites are situated in Changlinggang Forest Farm in Jianshi County, Enshi Autonomous Prefecture, Hubei Province, China, and Wanbaoshan Forest Farm in Longshan County, Xiangxi Autonomous Prefecture, Hunan Province, representing the central and southern regions of China, respectively. Established in 1957, Changlinggang Forest Farm stands as the largest Japanese larch base in southern China. Meanwhile, Wanbaoshan Forest Farm holds the distinction of being the largest Japanese larch base in Hubei Province. In 2000, Japanese larch was designated as the afforestation tree species for seedling cultivation and afforestation endeavors. The climate, terrain, altitude, and stand density of the two forest farms exhibit similarities and possess certain representativeness. Detailed stand data is illustrated in Figure 1.

### Experimental design and sampling collection

This study investigated a chronosequence of Japanese larch plantations. Soil samples were collected four stand-age stages, namely Young (J1, J2, J3), Middle-aged (M1, M2), Near-mature (N1, N2)and Mature stand (R1) to examine the responses of fungal communities to nitrogen and phosphorus metabolism during forest succession. The collection of samples follows the method of Zhang et al. (2022). Sample collection was conducted during the growing season within each forest stand. In each plot, areas with relatively homogeneous stand structure and similar site conditions were selected as sampling locations to minimize the influence of environmental heterogeneity. Plant samples included leaves, stems, and roots, which were collected using a random sampling approach within each plot. Soil samples were collected around the corresponding plants following an “S-shaped” pattern using a soil auger from the 0–20 cm surface layer. Visible stones, roots, and litter were carefully removed, and the soil was thoroughly homogenized to form one composite sample per replicate. We collected eight forest plot sample groups from four different forest ages, with three biological replicates for each sample group. After collection, all samples were immediately placed in pre-prepared sterile, sealed self-sealing bags. Soil samples were placed in insulated boxes with ice packs immediately after collection in the field to maintain a low temperature as much as possible and minimize changes in microbial activity. The samples were transported back to the laboratory on the same day, with transport time generally kept within 24 h. Upon arrival, soil samples were immediately pretreated: one portion was passed through a 2 mm sieve for physicochemical analyses, while another portion was stored at −20 °C for subsequent microbial analyses. Plant samples were washed, separated by tissue type, and either oven-dried or frozen for further analyses. Regarding sample amounts, approximately 10–20 g of soil was used for physicochemical property measurements, and about 5–10 g was used for microbial biomass and enzyme activity assays. For plant biomass and nutrient analyses, sample mass varied by tissue type, with 0.5–1.0 g (dry weight) used per sample. Independent replicates were established for each plot to ensure data representativeness and reliability. The sampling information is shown in Figure 1c. The collected rhizosphere soil samples were divided into three parts. One part was used to determine the basic physical and chemical properties of the soil, the other part was used to measure microbial biomass and soil enzyme activity, and the remaining part was used for Internal Transcribed Spacer (ITS) measurement analysis.

### Physiochemical analysis

Total porosity, capillary porosity, non-capillary porosity, and associated water-holding properties were assessed using the ring knife method (Yan et al., 2019). Hydrolyzable nitrogen (HN) was quantified employing the alkaline hydrolysis diffusion method, while total nitrogen was determined through Kjeldahl nitrogen determination (Clark et al., 2015). Available phosphorus was evaluated via sodium bicarbonate extraction and the molybdenum blue method, whereas available potassium was detected using ammonium acetate extraction (Zhu et al., 2016). Total phosphorus and total potassium were analyzed after digestion using an acidic mixture of HNO_3_-HCl-HF (4:1:1, v/v/v). The dried samples were pulverized and digested in an HNO_3_-HClO_4_ (3:1, v/v) mixture at 180 °C employing a graphite furnace (Hong et al., 2022). The resulting solutions were subsequently analyzed using inductively coupled plasma emission spectrometry. Microbial biomass nitrogen and microbial biomass phosphorus were determined via chloroform fumigation (Joshi and Garkoti, 2023). Soil acid phosphatase activity was assessed using the p-nitrophenyl phosphate method (Veloso et al., 2023), and urease activity was determined utilizing the sodium phenol-sodium hypochlorite colorimetric method (Guo et al., 2023).

### Statistical analysis

Samples were sequenced using ITS, and the raw data obtained were subjected to amplicon sequence variants (ASVs) clustering and annotation through sequence splicing and quality control (Fodelianakis et al., 2022). A rarefaction analysis was performed based on the ASVs clustering results to examine the sample sequencing coverage situation.

Alpha diversity calculations included Chao, Shannon, and Observed_species indices (Li et al., 2022). Chao and Observed species indices reflected community richness, Shannon index indicated community diversity. Species composition analysis integrated the community structure analysis of multiple samples, observing the community structure at different taxonomic levels, and was analyzed and visualized using the reshape2 and ggplot2 packages of R (v3.6.0). ASVs were taxonomically annotated at the 97% similarity threshold (García-López et al., 2021) using the software (QIIME v1.8.0 platform and Vsearch 2.7.1) to determine microbial community structure and composition at the phylum, genus, and ASV levels.

Beta diversity analysis assessed the similarity or difference in sample community composition (Crabot et al., 2020). Non-metric multidimensional scaling (NMDS) was employed as a data analysis method to reduce research objects (samples or variables) in multidimensional space for localization, analysis, and categorization while preserving the original relationships among the objects (Khodabandeh et al., 2023). Analysis and visualization were performed using the vegan and ggplot2 packages of R (v3.6.0).

Redundancy analysis (RDA) and variance partitioning analysis (VPA) were conducted using the vegan package of R. Variable inflation factor values (VIF) were calculated before performing redundancy analysis to eliminate redundancy factors with VIF > 12. Molecular ecological network (MENs) construction was based on the relative abundance of ASVs in different treatments (Wang X. et al., 2023). The network was built using Spearman’s test, with the top 20 genus-level results selected for correlation analysis. Critical values for species interactions were Spearman correlation > |0.6| and *p* < 0.05, used to filter out weakly correlated ASVs and reduce network complexity. All network analyses were conducted via the Molecular Ecological Network Analysis Pipeline (MENAP),^1^ with visualization performed using Gephi 0.9.1-beta. Intra-module connectivity (Zi) and inter-module connectivity (Pi) were calculated for each node based on differences in ASV abundance between modules in the coexistence network.

PICRUSt2 (Phylogenetic Investigation of Communities by Reconstruction of Unobserved States, v2) was used to infer potential functional profiles of microbial communities based on marker gene information, following the approach described by Douglas et al. (2018).

## Results

### Changes in soil physicochemical properties

#### Soil physicochemical properties of different forest stands

**Figure 2:**
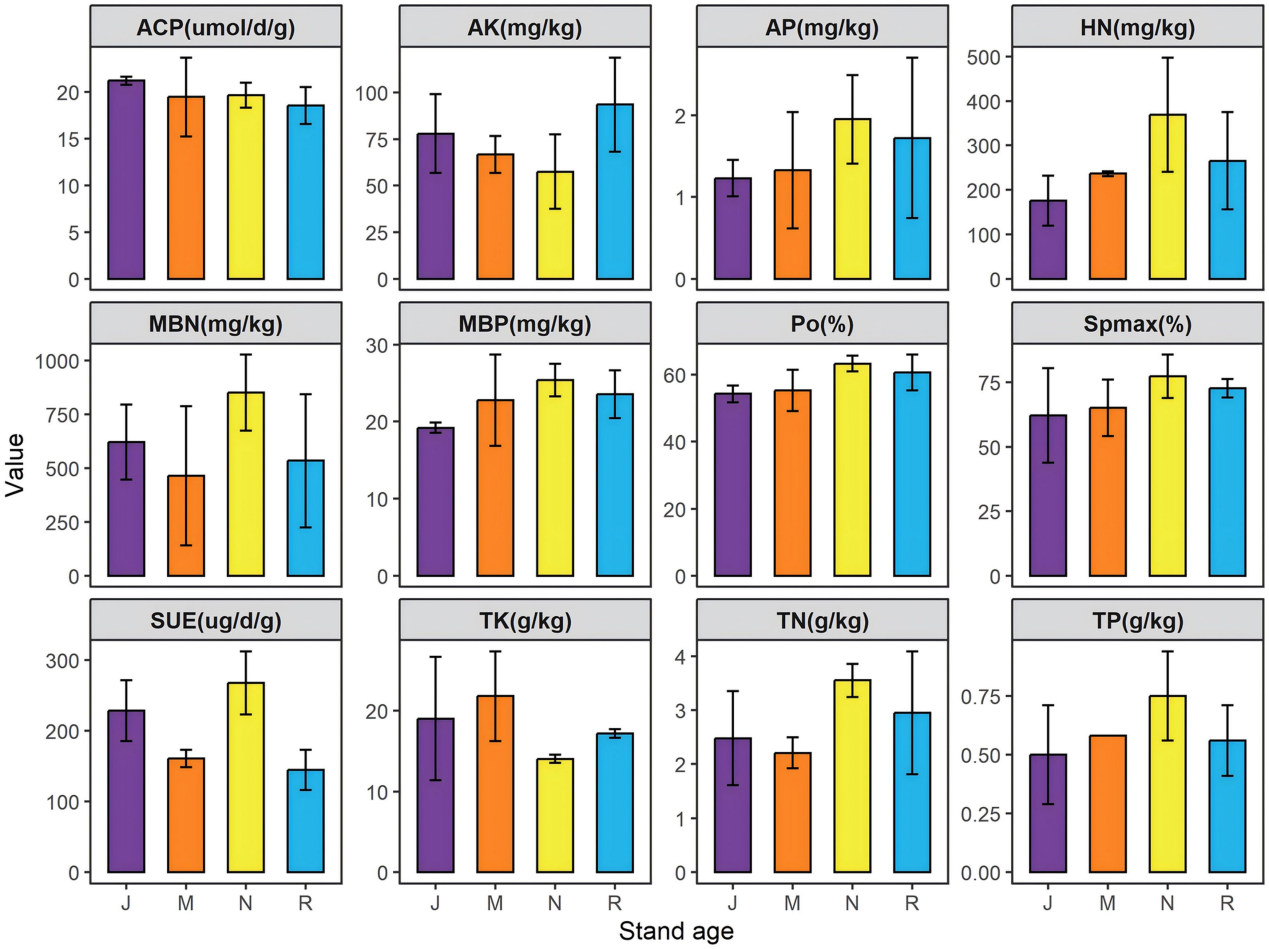
Soil physicochemical parameters of different sampling sites. J, Young stand; M, middle-aged stand; N, near-mature stand; R, mature stand; Sp_max_, Soil capillary water holding capacity; CPo, capillary porosity; NPo, non-capillary porosity; TN, total nitrogen; TP, total phosphorus; TK, total potassium; HN, hydrolyzable nitrogen; AP, effective phosphorus; AK, effective potassium; ACP, acid phosphatase; SUE, soil urease; MBN, microbial biomass nitrogen; MBP, microbial biomass phosphorus.

As forest age increased, soil capillary water holding capacity (Spmax) exhibited a pattern of initial increase followed by a decrease, peaking at 77.33% in the near-mature group (N). Similarly, the trends of capillary porosity (CPo) and non-capillary porosity (NPo) mirrored those of capillary water holding capacity. The peaks of total nitrogen (TN) and total phosphorus (TP) both occurred in the near-mature forest group (N), reaching 3.55 g/kg and 0.75 g/kg, respectively, with consistent trends. Total potassium (TK) reached its apex at 21.78 g/kg in the middle-aged forest group (M) before declining. Hydrolyzable nitrogen (HN) and available phosphorus (AP) exhibited a parallel increasing trend with TN and TP, subsequently declining after reaching peak levels in the near-mature forest group (N). Effective potassium (AK) did not display a significant trend with stand age and attained a maximum value of 99.44 mg/kg in the mature forest (R). Acid phosphatase (ACP) activity peaked in the young forest group (J), while soil urease (SUE) activity peaked in the near-mature forest group (N). Both microbial biomass nitrogen (MBN) and microbial biomass phosphorus (MBP) reached their highest levels in the near-mature forest group (Figure 2).

#### Correlations between N-P and environmental properties

Changes in environmental factors often lead to alterations in N and P metabolism, complicating the relationship between N-P metabolism and soil and microbial parameters. Correlation analysis involving soil physical factors (Spmax, CPo, NPo), soil nutrient factors (TN, TP, TK, HN, AP, AK), and microbial metabolism factors (ACP, SUE, MBP, and MBN) revealed several significant associations. TP exhibited a significant correlation with HN and NPo, and a strong correlation with TN. TN showed a significant correlation with MBN and NPo, while HN correlated significantly with AP. In contrast, no significant correlations were detected between acid phosphatase activity (ACP) and either TP or AP.

#### Alpha and beta diversity of different fungal communities

Sequence quality control involved filtering, merging, and removal of host plant gene sequences, resulting in a total of 1,658,272 clean reads with lengths ranging from 200 to 320 bp. These sequences were clustered into 2,860 ASVs, with a range of 409 to 882 ASVs in a single sample, reflecting considerable variation in fungal community composition among samples (Supplementary Figure 1).

**Figure 3:**
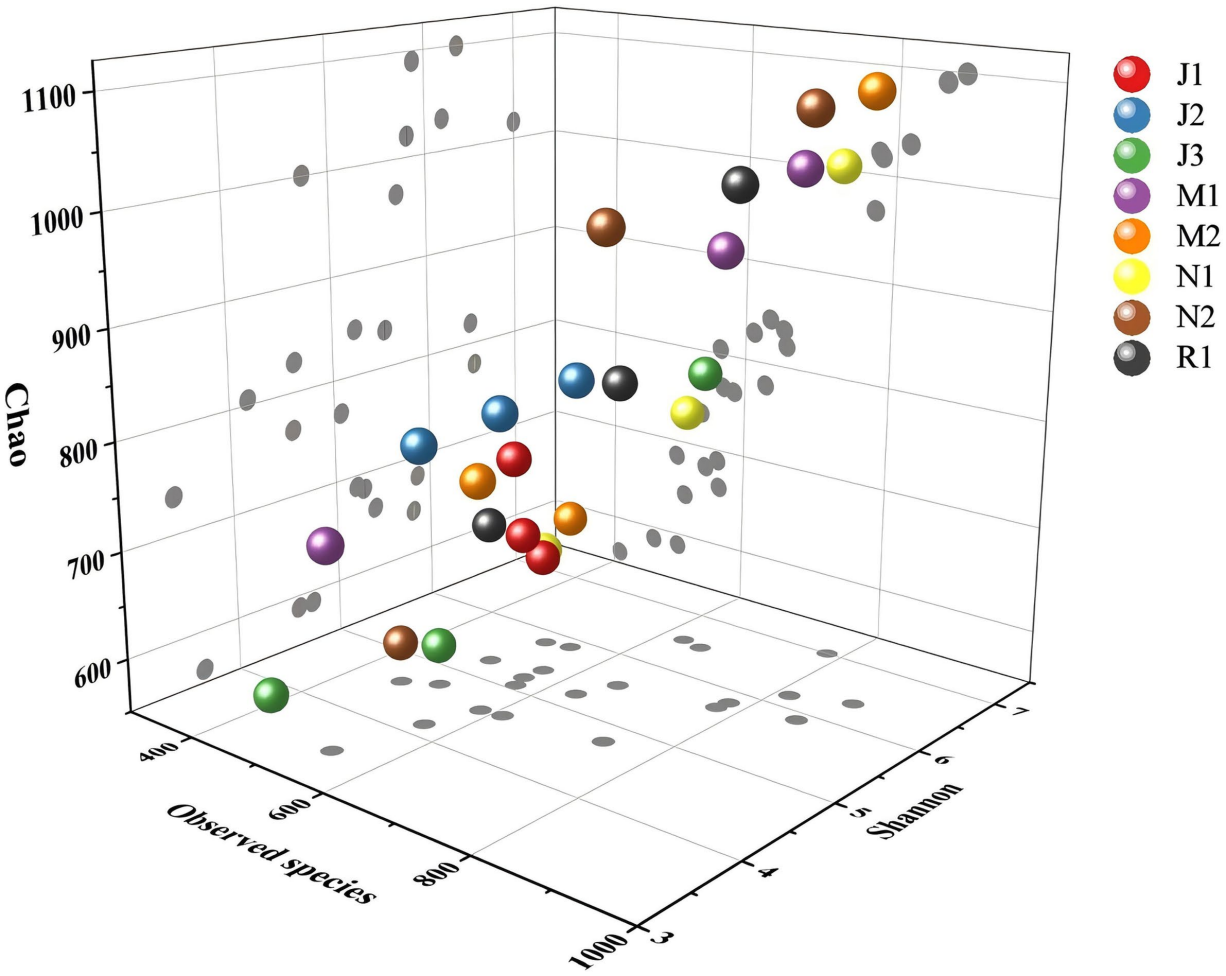
Alpha diversity indexes of different fungal communities. Observed species: No. of species actually observed. Shannon: Reflects community diversity index. Chao: Species abundance.

Alpha diversity was assessed at the ASV level using Observed species, Coverage, Shannon diversity, and Chao richness indices. As depicted in Figure 3, the number of observed species increased with tree age and then stabilized, with the highest values observed in the middle-aged forest group (M1) and the near-mature forest group (N2), while the lowest value was observed in the young forest group (J3). Most samples exhibited high coverage, with values ranging from 0.99 to 1, indicating good sequencing depth. The Shannon index, representing microbial community diversity, displayed a similar increasing-then-decreasing trend with tree age, while the Chao index, indicative of microbial community richness, demonstrated a gradual increase with tree age.

**Figure 4:**
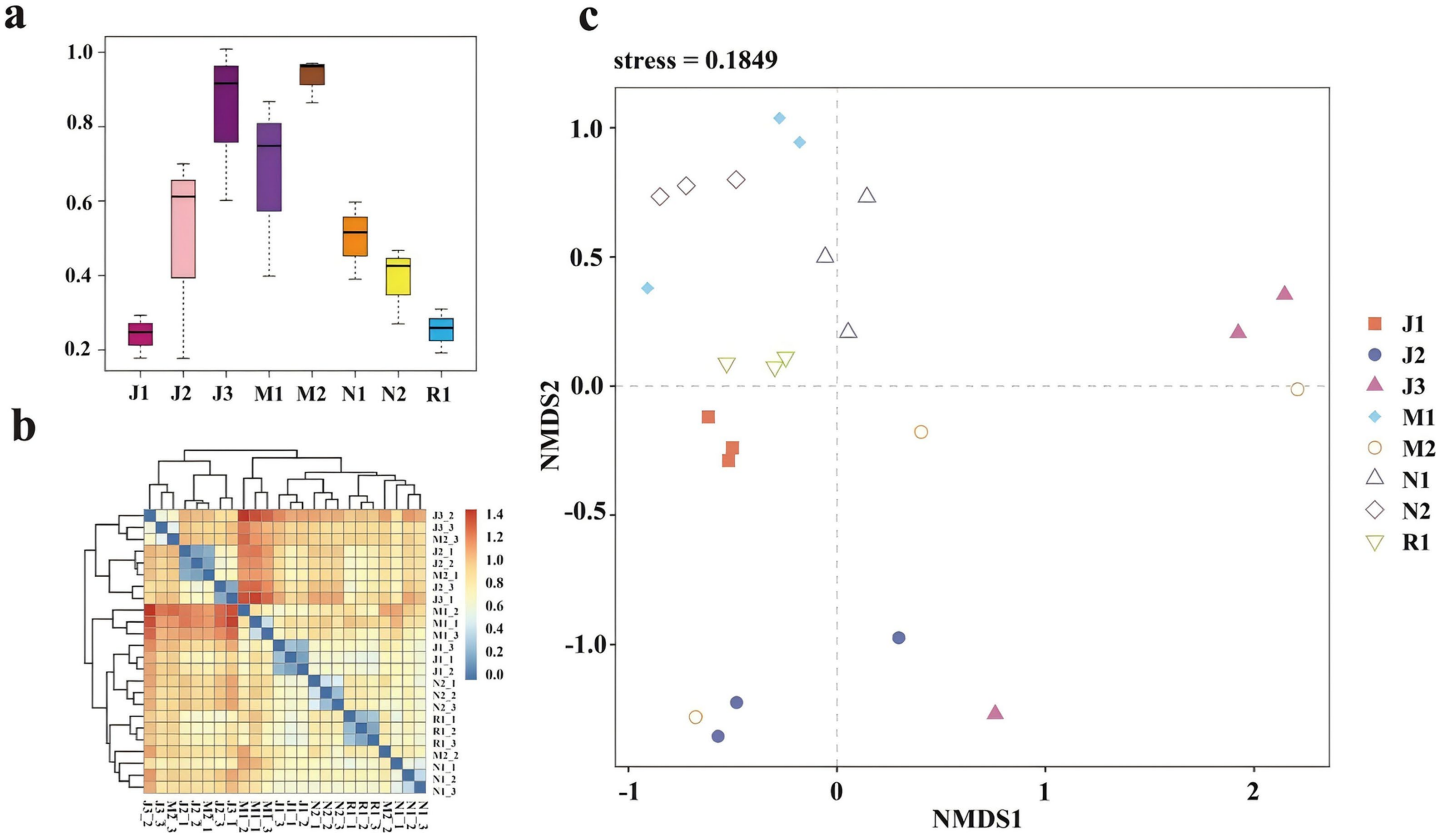
Weighted unifrac distance between different samples shown by boxplot **(a)** and heatmap **(b)**. The samples are color-coded according to the similarities between each of them: from zero (blue) to over (red). Non-metric multidimensional scaling (NMDS) **(c)**.

Beta diversity of the Fungal community was further elucidated through Unifrac distance (Figures 4a,b). Distances between fungal communities in young forests (J1, J2, and J3) were greater than those between fungal communities in near-mature forests (N1, N2) and mature forests (R1), indicating greater variation within the young forest group. Principal component analysis (Figure 4c) provided a clearer understanding of similarities and dissimilarities between microbial community structures by visualizing the distribution of sample points on corresponding axes. Tree age influenced the structure of the fungal community to some extent, as sample points clustered by age group. Near-mature groups N1 and N2 clustered closely, while mature (R1) samples formed a tight cluster. Conversely, juvenile groups J1, J2, and J3 were distributed separately, highlighting greater variability in the structure of microorganisms in young forests compared to near-mature age groups.

#### Fungal community composition and dominant species of different samples

**Figure 5:**
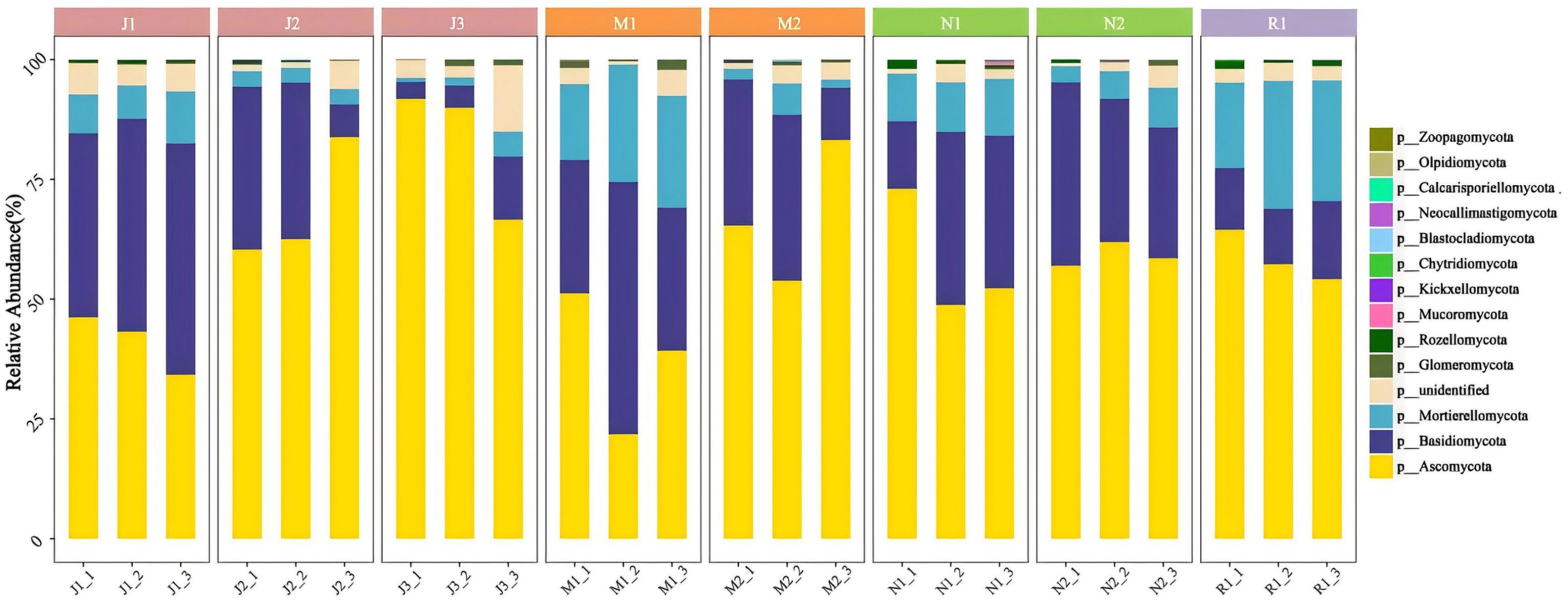
Species composition of different groups at phylum level. The horizontal axis represents the sample name, and the vertical axis represents the relative abundance of the species in the sample. The figure shows species information with relative abundance of over 1%.

To further elucidate the community structure and composition of fungal communities, the obtained ASVs from the samples underwent statistical analysis, revealing a total of 14 phyla with a relative abundance greater than 1%. Ascomycota exhibited the highest abundance across the samples, averaging at 59.18%. Basidiomycota also emerged as a dominant phylum, with an average share of 26.28%. Despite the prevalence of these phyla across all samples, differences in community structure were noted among various forest stages (Figure 5).

It was observed that within the young forest group, there was a notable increasing trend in relative abundance of Ascomycota and a decreasing trend in species abundance of Basidiomycota as tree age increased (J1-3). In comparison with young forests, the species abundance of Ascomycota decreased, while that of Basidiomycota significantly increased in the middle-aged (M) and near-mature forest stages (N). However, Ascomycota still accounted for the highest proportion. Notably, the species abundance of Mortierellomycota was markedly higher in mature forests compared to near-mature forests, surpassing that of Basidiomycota, which was more dominant in the near-mature forest stage. Changes in tree age influenced the microbial community structure and abundance to a certain extent, with the microbial community structure in young (J1, J2, J3) and mature forests (R1) exhibiting comparatively more stable structural patterns than those observed at intermediate stages.

#### The relationship between soil physical, soil nutrient levels, and microbial metabolic activities

The investigation of environmental factors aids in elucidating variations among environmental drivers of fungal community variation. Variance partitioning analysis (VPA) was conducted to categorize and assess the importance of 13 environmental factors on fungal community structure. These variables were grouped into three categories: soil physical factors (Spmax, CPo, and NPo), soil nutrient factors (TN, TP, TK, HN, AP, and AK), and microbial metabolic factors (ACP, SUE, MBN, and MBP). 10 environmental factors were selected for Redundancy Analysis (RDA) after eliminating correlated variables (TN, TP, and TK) based on variance inflation factors (VIF > 12).

**Figure 6:**
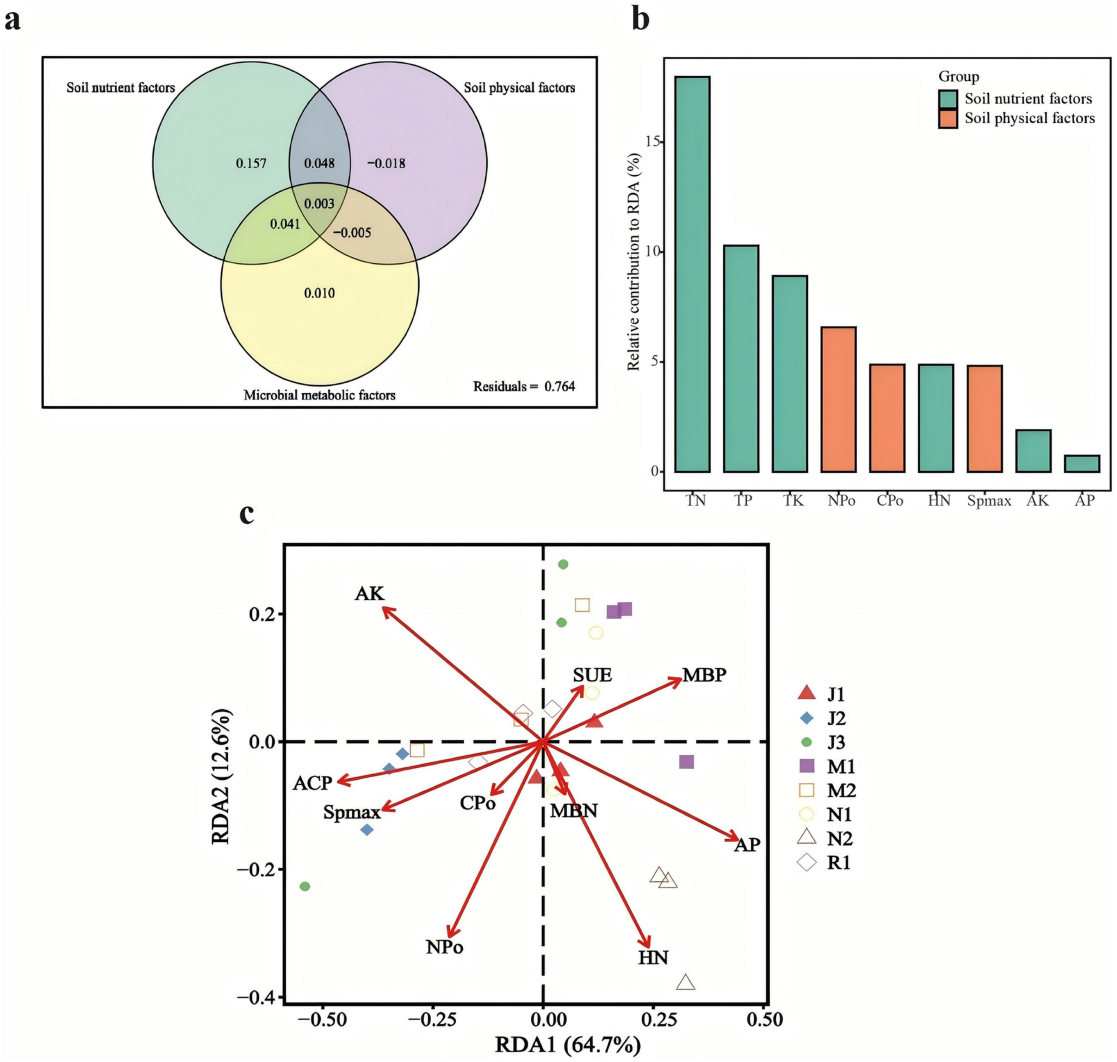
Variance partitioning analysis [VPA, **(a)**]. Contribution of microbial metabolic variables to RDA axes **(b)**. Redundancy analysis [RDA, **(c)**]. Variables presented in RDA plot were selected by variance inflation factors. For VPA, variables presented in RDA were separated into three groups: soil physical factors (Spmax, CPo, and NPo), soil nutrient factors (TN, TP, TK, HN, AP, and AK), and microbial metabolic factors (ACP, SUE, MBN, and MBP).

Figure 6a illustrates that soil nutrient factors independently accounted for 15.73% of the variance in fungal community structure, exerting the primary influence compared to soil physical factors and microbial metabolism factors. The RDA plot in Figure 6c reveals significant correlations between near-mature forests and soil nutrient factors (AP, HN) as well as microbial metabolism factors (MBN and MBP). To further quantify the contribution of individual environmental variables in the RDA model, the marginal effects of each factor were calculated and visualized as a bar chart (Figure 6b).

### Molecular ecology networks and keystone taxa

#### Topological properties of different MENs

**Figure 7:**
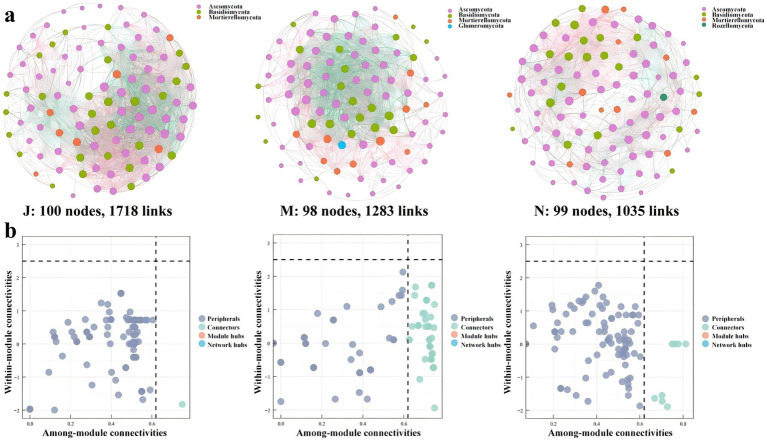
Co-occurrence network of microbial communities in each sample and node classification **(a)**. The size of the dots represents the magnitude of abundance, and the thickness of the line represents the magnitude of correlation; the color of the dots represents the phylum to which they belong, with a red line indicating positive correlation and a blue line indicating negative correlation. The Zi-Pi diagram showed the distribution of ASVs according to their Zi and Pi values based on the topological structure **(b)**. Network hubs: Zi > 2.5, Pi > 0.62; module hubs: Zi > 2.5, Pi ≤ 0.62; connectors: Zi ≤ 2.5, Pi > 0. 62; peripheral nodes: Zi ≤ 2.5, Pi ≤ 0.62.

MENs analysis unveils species interactions and structural stability within groups. Three MENs were constructed based on the ASVs of each sample group, depicting interspecific correlations of microbial communities in young (J), middle-aged (M), and near-mature (N) forests (Figures 7a, b). Corresponding topological properties are outlined in Table 1. The networks were delineated with corresponding gates, retaining correlations with *p* < 0.05 and |R| > 0.6. Ascomycota, Basidiomycota and Mortierellomycota dominated the network nodes of J, M and N. Notably, Glomeromycota was additionally present in the network of the middle-aged forest (M), whereas Rozellomycota occurred exclusively in the near-mature forest (N). These networks exhibit typical hierarchical, small-world, and modular characteristics, facilitating subsequent molecular ecological network analysis. Results indicate that the MENs of J, representing young forests, exhibited more nodes and interspecific connections, with 1.34 to 1.65 times higher connections than M and N. M displayed the most modules, while N demonstrated the best modularity.

**Table 1:** Topological properties of the empirical MENs of microbial communities in J, M, and N groups.

Communities	J	M	N
No. of original ASVs	100	100	100
Similarity threshold (st)	0.6	0.6	0.6
Total nodes	100	98	99
Total links	1,718	1,283	1,035
Avg path length (GD)	1.941	1.925	2.084
Avg clustering coefficient (avgCC)	0.728	0.553	0.595
No. of modules	3	11	7
Modularity	0.31	0.184	0.383

#### Keystone taxa identification

Different species of nodes in MENs fulfill distinct ecological and functional roles (Deng et al., 2012), and identifying key taxa based on their within-module connectivity (Zi) and inter-module connectivity (Pi) values can elucidate the ecological niches and functions of fungi in various environments. ASVs in the network were categorized into four groups: network hubs (Zi > 2.5, Pi > 0.62), module hubs (Zi > 2.5, Pi ≤ 0.62), connectors (Zi ≤ 2.5, Pi > 0.62), and peripheral nodes (Zi ≤ 2.5, Pi ≤ 0.62) (Meng et al., 2023).

Results showed that most ASVs in each network were classified as peripheral nodes. Notably, no network hubs or module hubs were detected in J, M, and N networks. The number of nodes classified as connectors in J, M, and N networks was 1, 54, and 10, respectively. Phylogenetic classification of key taxa (Supplementary Table 1) indicated that species under the clades Ascomycota, Basidiomycota, Glomeromycota, and Mortierellomycota serve as key taxa in MENs. Co-occurrence network analysis revealed overlapping key taxa in J, M, and N. Ascomycota holds pivotal positions in all three network modules, underscoring its significant ecological niche.

#### Functional gene prediction and distribution

**Figure 8:**
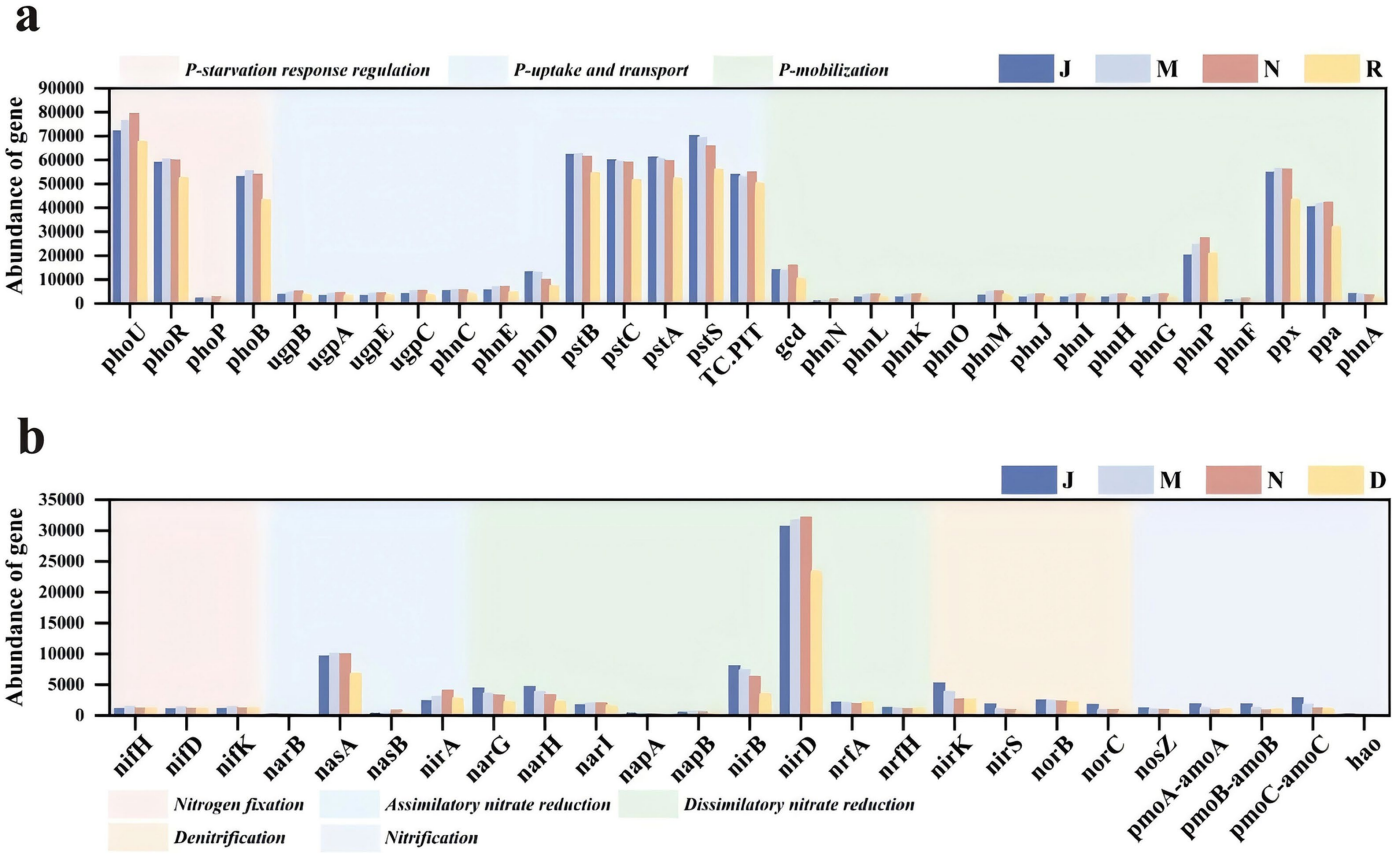
The abundance of soil microbial phosphorus cycle functional genes under different forest ages **(a)**. The phosphorus cycle consists of three parts: P-starvation, P-mobilization, P-uptake, and transport. The abundance of soil microbial nitrogen cycle functional genes under different forest ages **(b)**. The nitrogen cycle consists of five parts: nitrogen fixation, assimilatory nitrate reduction, dissimilatory nitrate reduction, denitrification, and nitrification.

To further elucidate the distribution of functional genes in different sample groups and their relationships with key ecological taxa, PICRUSt was employed to predict functional genes in the samples (Sun et al., 2020). Figure 8 presents the predicted KEGG Orthologs (KOs) and their corresponding gene abundances across different samples.

Microbial involvement in P cycling processes primarily encompasses phosphorus uptake and transport (P uptake and transport), phosphorus starvation response regulation (P starvation response regulation), and inorganic phosphorus solubilization and organic phosphorus mineralization (P mobilization) (Ibrahim et al., 2022; Sindhu et al., 2014). Among the functional genes associated with inorganic P solubilization and organic P mineralization, gcd, phnP, ppa, and ppx exhibited high gene abundance, with ppx displaying the highest abundance. The abundance of gcd, phnP, and ppa genes peaked in the near-mature (N) forest and decreased as the forest aged into the mature (R) stage. The gene ppx exhibited the highest abundance in the middle-aged (M) forest and the lowest in the mature (R) forest. Functional genes such as phoB, phoR, and phoU were prevalent in the middle-aged (M) and mature (R) forests. Genes involved in the regulation of P starvation response, such as phoB, phoR, and phoU, displayed high abundance, with phoU exhibiting the highest abundance. The abundance of phoU increased gradually with stand age, peaking in the near-mature forest (N), and declining in the mature forest (R). Conversely, the gene abundance of phoB and phoR peaked in the middle-aged forest (M) and gradually decreased as the forest aged, with the lowest abundance observed in the mature forest (R). Functional genes involved in P uptake and translocation, such as pstA, pstB, pstC, pstS, and Pit, exhibited higher abundance, with pstS displaying the highest abundance. The gene abundance of pstA, pstB, and pstS showed a gradual decreasing trend with forest age, with the lowest abundance observed in the mature forest stage (R).

N cycling processes mediated by microorganisms encompass biological N fixation, ammonification, anaerobic ammonia oxidation, nitrification, denitrification, and dissimilatory nitrate reduction to ammonium (Liu Y. et al., 2024; Wang X. W. et al., 2023). During assimilatory nitrate reduction, nasA and nirA exhibited relatively high gene abundances, with nasA showing the highest abundance. The abundance of nasA peaked in the middle-aged forest stage (M) and was significantly lower in the mature forest stage (R) compared with other stages. In contrast, nirA showed a gradual increase in abundance with stand age, reaching its maximum in the near-mature forest (N) and declining in the mature forest (R), with the lowest abundance observed in the young forest stage (J). In the N fixation process, the abundances of nifD, nifK, and nifH did not differ significantly among stand ages; however, all three genes peaked in the middle-aged forest stage (M) and exhibited the lowest abundances in the young forest (J). During dissimilatory nitrate reduction, nirD exhibited markedly higher abundance than other related functional genes. Its abundance increased with stand age, peaking in the near-mature forest (N), followed by a decline in the mature forest (R). In the nitrification process, pmoC-amoC functional genes showed relatively high abundance, which decreased progressively with increasing stand age. Its abundance decreased with aging. During denitrification, nirK gene abundance was highest in the juvenile (J) stage and lowest in the mature (R) stage among the forest ages (Figure 8).

## Discussion

### Effects of tree age on N and P turnover in plant rhizosphere soil

Changes in tree growth and stand age are key factors affecting N and P nutrient accumulation (Wu et al., 2020). The biomass produced during tree growth affects soil N input and P turnover (Ehtesham and Bengtson, 2017). In order to evaluate the effects of tree age on N and P nutrients, we studied growth-related soil physical and chemical properties, and microbial metabolic activities at different tree ages. It was found that the soil N and P elements and their effective states reached the peak in the near-mature stage of the forest (N), and gradually decreased and stabilized in the mature stage of the forest (R) (Figure 1). The variation trends of TP and TN showed similar trends in previous studies (Deng et al., 2019; Wu et al., 2020). The TN in the rhizosphere soil of larch in Northeast China showed an increasing trend in different time series, reaching the peak at the mature stage of forest, while TP showed a decreasing trend, reaching the minimum value of 0.49 g/kg at the near mature stage of forest (Yan et al., 2017). Inconsistent trend of nitrogen and phosphorus nutrient content may be caused by regional and tree species differences (Chen et al., 2016). Compared with the young forest stage (J), the values of soil TN, TP, HN, and AP in the near-mature forest stage (N) increased by 43.1, 50.0, 109.3, and 58.5%, respectively in our study. The nutrient demand of different growth stages (J-R) varied (Jiang et al., 2017).

In this study, the soil TN concentration was between 2.21 to 3.55 g/kg, and the TP content was between 0.5 to 0.75 g/kg (Figure 1). Soil N: P reflects the balance of N and P during plant growth and development, and can indicate the supply of soil nutrients during plant growth (Zhao et al., 2015). In our study, the N: P ratios of rhizosphere soil at different forest ages were 4.96 (J), 3.81 (M), 4.73 (N) and 5.26 (R), which were lower than the national soil N:*p* (5.20) level in the early stage of forest maturity (Sun et al., 2024). This indicates that the soil nitrogen content is generally low, and the growth may be inhibited by nitrogen. In the mature stage of the forest (R), the soil phosphorus content decreases, and the trees are limited by phosphorus. Soil N: P showed a trend of decreasing first and then increasing, which was consistent with the trend of N: P ratio in rhizosphere soil of *Pinus massoniana* Lamb. plantation in southern China (Xu et al., 2022).

Microorganisms are the main driving forces to assist plants in absorbing nitrogen and phosphorus nutrients from the soil (Cao et al., 2022; Peng et al., 2022). In detail, plant N and P uptake is mediated by specific transporters, such as nitrate transporters (NRTs) and phosphate transporters (PHTs), which involved various nutrient signal transduction, gene regulation, and morphological and physiochemical changes that may recruit beneficial microorganisms and reallocate their structures and functions to help the plant for more intelligent and precise nutrient uptake (Oldroyd and Leyser, 2020). The analysis of microbial biomass in this study showed that with the increase of forest age, the content of MBP increased first and then decreased, which reflected the trend of TP and AP in rhizosphere soil of different forest ages. It reached the maximum value of 25.39 mg/kg in near mature forest stage (N), which was 32.1% higher than that in young forest stage (J). MBP is a dynamic phosphorus pool that regulates the availability and turnover of phosphorus (Peng et al., 2022). Similarly, the MBN content of different forest ages reflected the change trend of TN and SUE. The MBN value was the highest in the near-mature forest stage (N), reaching 850.56 mg/kg, which was 36.9% higher than that in the young forest stage (J). Microorganisms can help plants acquire these key nutrients more effectively through nitrogen fixation, phosphorus and potassium solubilization (Chang et al., 2024; Philippot et al., 2023; Zhu et al., 2023). For example, phosphate-solubilizing bacteria can secrete organic acids and phosphatases to improve the bioavailability of phosphorus and regulate the conversion process of phosphorus (Li H.-Z. et al., 2024); nitrogen-fixing bacteria can produce nitrogenase to convert atmospheric nitrogen into ammonia (Sankari et al., 2022). Collectively, these microbial-mediated processes highlight the potential role of soil microorganisms in supporting plant nitrogen and phosphorus nutrition.

### Effects of tree age on microbial community structure in rhizosphere soil

Increasing forest age is generally associated with changes in soil organic matter (SOM), soil nutrient availability, and microbial abundance in the rhizosphere, which may be partly related to age-related differences in tree growth and litter inputs. Root exudates are an important form of communication between plants and microorganisms, and can also microorganisms that are potentially involved in soil nutrient transformations relevant to plant nutrient acquisition (Bai et al., 2022). For example, flavonoids in maize root exudates recruit rhizosphere oxalate bacteria that can promote N utilization and maize lateral root growth (Yu et al., 2021). Increased microbial biomass and enzyme activities may reflect enhanced microbial involvement in soil nutrient transformation processes in the rhizosphere (Figure 1).

Tree age was significantly associated with changes in the diversity of plant rhizosphere microbial communities. Alpha diversity analysis showed that the diversity and abundance of fungal communities gradually increased and then decreased with changes in forest age (Figure 3). A similar pattern has been reported in Korean pine forests, where soil fungal diversity exhibited an increase followed by a decrease along forest development stages (Han et al., 2016). Litter input is limited, which may lead to low microbial community diversity. Similar to previous results (Hu et al., 2022), as larch stands develop, soil nutrient availability is improved and soil microbial diversity is also increased.

Tree age was an important factor associated with variations in plant rhizosphere microbial community structure. The structure of the microbial community varied among forest age stages, and the fungal community was mainly composed of Ascomycota, Basidiomycota and Mortierellomycota (Figure 5). Other studies have also confirmed this result (Liu et al., 2020; Sui et al., 2022; Wang et al., 2022a). Most Ascomycota, Basidiomycota and Mortierellaomycota are predominantly saprotrophic fungi. They are key decomposers of forest plant biomass, which can decompose forest litter and increase SOM content (Büttner et al., 2021; Manici et al., 2024; Salo et al., 2019). Microorganisms convert organic nitrogen into inorganic forms by decomposing organic matter available for plant uptake. The input of organic matter can also change the composition of genes related to the phosphorus cycle and promote the transformation of phosphorus (Huang et al., 2024). In addition, NMDS analysis showed that the rhizosphere microbial community of the forest changed greatly in the early stage of development, and tended to gather when the forest was close to maturity (Figure 4c). The soil fungal community structure is related to the change of soil nutrient accumulation (Li T. et al., 2023). The soil nutrient accumulation tends to be stable when the forest is nearly mature, and the number of microbial species is also relatively stable (Figure 3).

Key taxa occupying the core niches of microbial communities play crucial roles in shaping community structure and function. Molecular ecological network (MENs) analysis across different forest age stages revealed that Ascomycota was the dominant phylum shared among all three networks (Supplementary Table 1). Basidiomycota and Mortierellomycota were the dominant phyla in the molecular ecological network of middle-aged forest (M), and the abundance of key taxa in the MENs was higher in the middle-aged forest (M). Ascomycota was not only the key taxa of the important niches of MENs, but also the dominant species of each forest age sample, with an average percentage of 59.18% (Figure 5). Ascomycota is involved in the decomposition of organic matter and the assimilation of root exudates (Hugoni et al., 2018; Wang et al., 2022b). In this study, the abundance of Basidiomycota in J1-J3 decreased and the abundance of Ascomycota increased (Figure 5). It was reported that the increase of litter was beneficial to the transfer of Basidiomycota to Ascomycota (Dang et al., 2017). With the increase of forest age, SOM was accumulated due to the increase of diversity of litter and understory vegetation (Wan et al., 2023), but the ability of Ascomycota to degrade lignin was limited (Osono, 2007). Basidiomycota dominated the decomposition of litter in the middle and late stages. It can produce a variety of extracellular oxidases (Baldrian, 2006) and degrade recalcitrant lignin compounds (Lundell et al., 2010; Osono, 2007). Ascomycota and Basidiomycota are important groups for ectomycorrhiza (ECM) formation. Ectomycorrhizal fungi (ECMF) regulate soil nutrients through a variety of mechanisms, such as enhancing soil phosphorus bioavailability, promoting SOM decomposition and improving soil microbial activity (Nicolás et al., 2019; Qi et al., 2022). Interestingly, Glomeromycota, which forms arbuscular mycorrhiza (AM), became a key species in M (Supplementary Table 1). Arbuscular mycorrhizal fungi (AMF) can recruit microbial communities, stimulate their nitrogen and phosphorus turnover function, and improve N and P utilization (Wang G. et al., 2023; Zhang J. et al., 2024).

### Effects of plant-microbiome interaction on N and P nutrient metabolism

Rhizosphere microorganisms are the key driving factors between soil and forest nitrogen and phosphorus cycles (Cui et al., 2018; Zhang Y. et al., 2024). Their composition and functions are affected by the regulation of tree growth and soil properties, and can affect the circulation of nitrogen and phosphorus nutrients in the rhizosphere, which in turn participates in and regulates the turnover of nitrogen and phosphorus in plants and soils. Microbial functional genes are mainly involved in biochemical processes and mediate element cycling by encoding specific functional enzymes and metabolites. Their abundance can predict the biogeochemical cycling potential and metabolic characteristics of soil microorganisms (Tu et al., 2017). The results of this study showed that the microbial community-mediated N and P cycle processes were related to plant age (Figure 8), and the abundance of soil microbial functional genes also changed with forest age (Li Y. et al., 2024).

**Figure 9:**
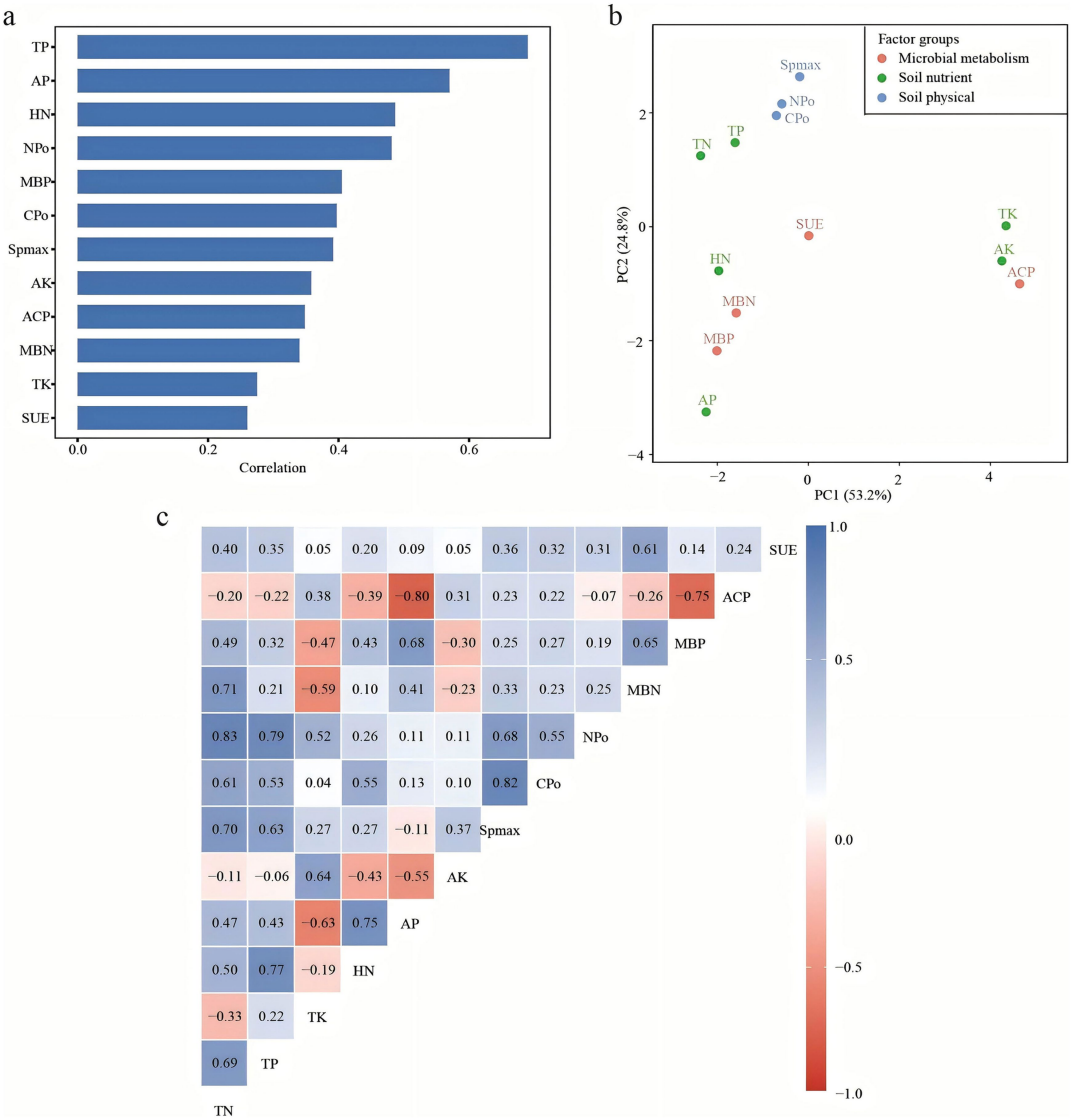
Absolute correlation strength among soil physicochemical and microbial variables **(a)**. Principal component analysis of soil properties and microbial indices based on correlations **(b)**. Correlation analysis of soil parameters **(c)**. The correlation coefficient is obtained by calculating the Pearson’s correlation coefficient, which is used to reflect the interrelationship between the variables.

In this study, there was a significant positive correlation between MBN and TN (*p* ≤ 0.05) (Figure 9), and MBN at different forest ages also reflected the changing trend of TN and SUE (Figure 1). When the soil TN content was the lowest in the middle-aged forest (Figure 1), we found that in the process of nitrogen cycle, the expressions of nitrogen fixation (nifH, nifD and nifK) and assimilative nitrate reduction (nasA) functional genes were more abundant in the middle-aged forest (M) (Figure 8b). As trees grow from young to middle-aged, tree growth is more dependent on nitrogen uptake to meet their nitrogen needs (Sun et al., 2016). Soil nitrogen storage is reduced, and microorganisms accelerate biological nitrogen fixation and establish symbiotic or reciprocal relationships with plant roots, thereby promoting root expansion and nutrient uptake (Islam et al., 2013; Li P. et al., 2024). Microorganisms are a key link in driving nitrification and denitrification and regulating soil nitrogen availability and N_2_O emissions (Song and Niu, 2022). The abundance of functional genes related to nitrification process (nosZ, pmoA-amoA, pmoB-amoB, pmoC-amoC and hao), denitrification process (nirK, nirS, norB and norC) and dissimilatory nitrate reduction processes (narG, narH and nirB) was the highest in the young forest period (Figure 8b). Young trees grow faster and have higher nitrogen requirements, resulting in more nitrogen uptake (Sun et al., 2016). The interaction between plants and rhizosphere microorganisms affects the physical and chemical properties of the soil, shapes the rhizosphere microbial community, activates nitrogen cycle genes, and promotes nitrogen fixation and transfer (Dong et al., 2024).

There was a significant negative correlation between MBP and ACP, as well as between AP and ACP (*p* ≤ 0.05) (Figure 9), which was consistent with previous observations (Hofmann et al., 2016). The contents of AP and MBP were the lowest, while the activity of ACP was the highest, which was 21.21 umol d^−1^ g^−1^ (Figure 1). ACP activity is one of the main factors affecting MBP (Chen et al., 2017). It has been reported that ACP activity is affected by plant phosphorus starvation. The degree of phosphorus limitation in soil and the availability of organic phosphorus compounds (He et al., 2024)could induce microbial ACP secretion to mobilize organic phosphorus in low-phosphorus soils (Ma et al., 2021). AP and MBP showed an increasing trend with the increase of forest age (Figure 1), and the increase of soil available phosphorus could be attributed to the utilization of mineral phosphorus by microorganisms. Microorganisms can dissolve insoluble inorganic phosphorus and mineralize more organic phosphorus than they need to be fixed in biomass and provide soluble inorganic phosphorus to plants (Chen et al., 2021; Hofmann et al., 2016). The increase in soil AP may be due to the increase in gene abundance that controls soil microbial inorganic phosphorus solubilization and organic phosphorus mineralization (Zhi et al., 2023). During the phosphorus cycle, the abundance of functional genes related to microbial phosphorus starvation response and phosphorus mobilization regulation increased with the increase of forest age, reaching a peak in the near-mature forest stage. The abundance of phosphorus starvation genes (PhoU, PhoR and PhoB), phosphorus mobilization genes (gcd, phnP, ppx, ppa) and phosphorus uptake and transport (pstB, pstC, pstA, pstS and Pit) were much higher than other genes in the same group (Figure 8a). Inorganic phosphorus dissolution and organic phosphorus mineralization related genes gcd, ppx, ppa and PhnP dominated the increase of soil phosphorus utilization capacity (Zhi et al., 2023). The genes (phoU, phoR and phoB) involved in the regulation of phosphorus starvation response enable microorganisms to utilize external phosphorus sources. These genes are closely related to genes involved in phosphorus uptake and transport (such as pst) and control the expression of alkaline phosphatase-encoding genes (Hsieh and Wanner, 2010).

## Conclusion

This study investigated the dynamics of soil nitrogen (N) and phosphorus (P) nutrients across different tree ages and provided evidence for associations between fungal communities and N and P cycling. The progressive increase in tree biomass in larch plantations, from juvenile to mature stages, was associated with changes in nutrient inputs, coincided with shifts in soil N and P distribution, was linked to variations in microbial biomass and metabolic potential. Collectively, the characterization of microbial functional genes and key taxa provides valuable insights into the mechanisms underlying forest nutrient cycling and offers a scientific basis for enhancing forest productivity and regulating nutrient recycling in forest ecosystems.

## Data Availability

The datasets presented in this study can be found in online repositories. The names of the repository/repositories and accession number(s) can be found in the article/Supplementary material.

## Footnotes

1
http://129.15.40.240/MEAN/

## Glossary

N: Nitrogen
P: Phosphorus
ITS: Internal transcribed spacer
ASVs: Amplicon sequence variants
NMDS: Non-metric multidimensional scaling
Spmax: Soil capillary water holding capacity
CPo: Capillary porosity
NPo: Non-capillary porosity
TN: Total nitrogen
TP: Total phosphorus
TK: Total potassium
HN: Hydrolyzable nitrogen
AP: Effective phosphorus
AK: Effective potassium
ACP: Acid phosphatase
SUE: Soil urease
MBN: Microbial biomass nitrogen
MBP: Microbial biomass phosphorus
VPA: Variance partitioning analysis
RDA: Redundancy analysis
VIF: Variable inflation factor values
MENs: Molecular ecological network
GD: Avg path length
avgCC: Avg clustering coefficient
KOs: KEGG orthologs
SOM: Soil organic matter
ECM: Ectomycorrhiza
ECMF: Ectomycorrhizal fungi
AM: Arbuscular mycorrhiza
AMF: Arbuscular mycorrhizal fungi
