# Supplementary material for: Soil N and P nutrient metabolism affected by fungal community in larch plantation
Source: Front Microbiol. 2026 Mar 23;17:1658803. doi: 10.3389/fmicb.2026.1658803 (PMC13052267; doi:10.3389/fmicb.2026.1658803)
Supplement: Supplementary file 1 [file Table_1.docx]

**Supplementary Material**

1. **Supplementary Figures and Tables**
   1. **Supplementary Figures**


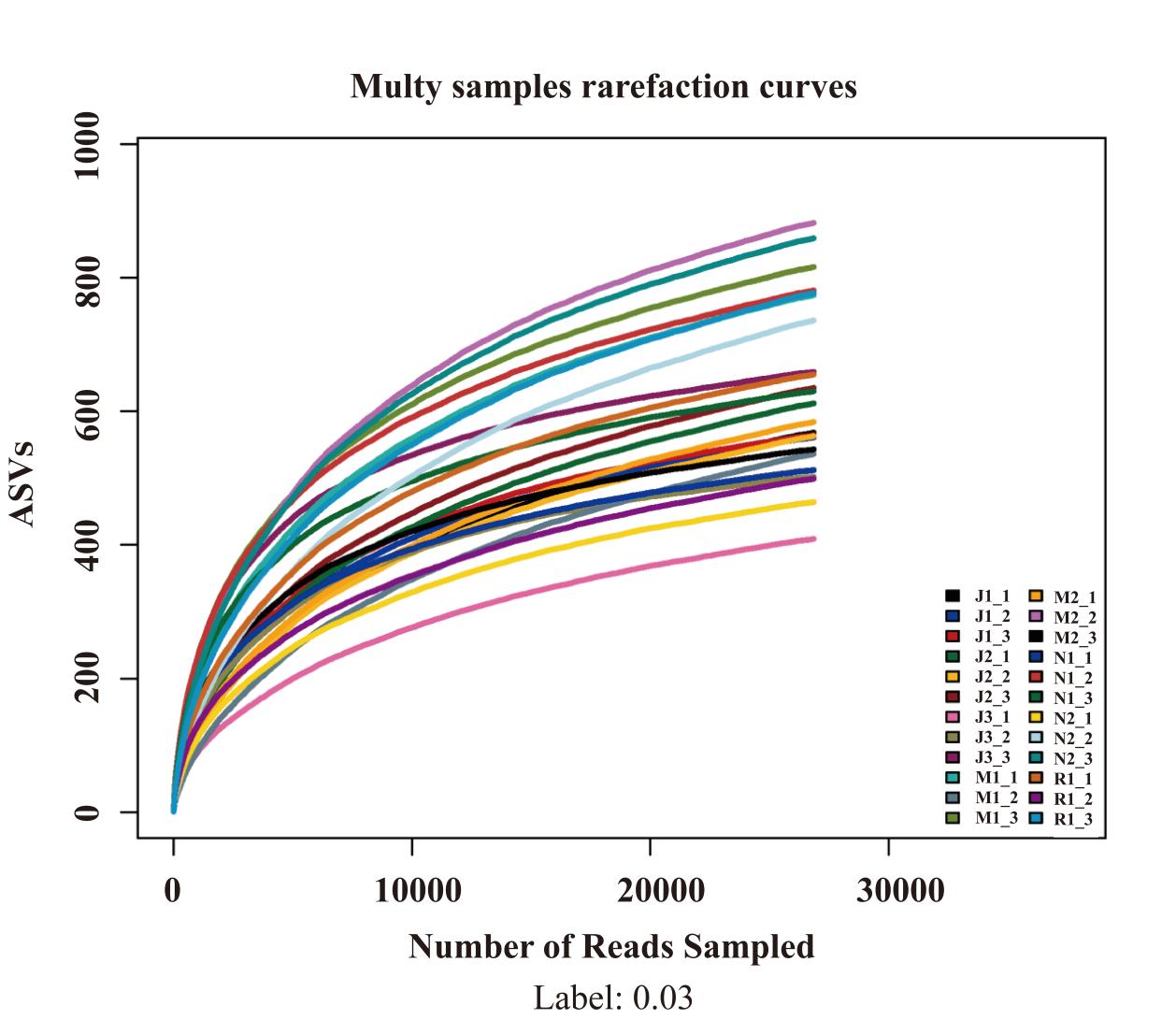


**Supplementary Figure 1** Rarefaction curves of each sample based ITS sequences

- 1. **Supplementary Table**

**Supplementary Table 1** . Affiliation of keystone taxa in Zi-Pi graph. P: Phylum, C: Class, F: Family ,O: Order, G: Genus and S:Species.

|  |  | **Nodes** | **affiliations** | **Phylum** |
| --- | --- | --- | --- | --- |
| J | connectors | ASV_20 | S:*Archaeorhizomyces_sp* | Ascomycota |
| M | connectors | ASV_103 | S:*Pneumocystis_jirovecii* | Ascomycota |
|  |  | ASV_117 | F:Pezizaceae |  |
|  |  | ASV_119 | P:Ascomycota |  |
|  |  | ASV_123 | S:*Archaeorhizomyces_sp* |  |
|  |  | ASV_127 | S:*Archaeorhizomyces_s*p |  |
|  |  | ASV_128 | S:*Archaeorhizomyces_sp* |  |
|  |  | ASV_13 | S:*Archaeorhizomyces_sp* |  |
|  |  | ASV_144 | S:*Archaeorhizomyces_sp* |  |
|  |  | ASV_167 | S:*Archaeorhizomyces_sp* |  |
|  |  | ASV_169 | S:*Penicillium_sp* |  |
|  |  | ASV_176 | P:Ascomycota |  |
|  |  | ASV_18 | P:Ascomycota |  |
|  |  | ASV_206 | O:GS33 |  |
|  |  | ASV_213 | S:*Fusarium_oxysporum* |  |
|  |  | ASV_223 | P:Ascomycota |  |
|  |  | ASV_243 | S:*Sarcopeziza_sicula* |  |
|  |  | ASV_270 | S:*Ramophialophora_sp* |  |
|  |  | ASV_30 | S:*Archaeorhizomyces_sp* |  |
|  |  | ASV_40 | S:*Archaeorhizomyces_sp* |  |
|  |  | ASV_41 | C:Archaeorhizomycetes |  |
|  |  | ASV_46 | S:Tuber_sp |  |
|  |  | ASV_49 | C:Archaeorhizomycetes |  |
|  |  | ASV_5 | S:*Penicillifer_pulcher* |  |
|  |  | ASV_59 | S:*Atractospora_decumbens* |  |
|  |  | ASV_60 | P:Ascomycota |  |
|  |  | ASV_63 | F:Nectriaceae |  |
|  |  | ASV_72 | F:Pyronemataceae |  |
|  |  | ASV_79 | S:*Cryptosporiopsis_sp* |  |
|  |  | ASV_8 | S:*Archaeorhizomyces_sp* |  |
|  |  | ASV_85 | S:*Kodamaea_ohmeri* |  |
|  |  | ASV_90 | S:*Acrophialophora_fusispora* |  |
|  |  | ASV_99 | S:*Atractospora_decumbens* |  |
|  |  | ASV_103 | S:*Pneumocystis_jirovecii* |  |
|  |  | ASV_117 | F:Pezizaceae |  |
|  |  | ASV_119 | P:Ascomycota |  |
|  |  | ASV_1  ASV_110  ASV_137  ASV_180  ASV_183  ASV_188  ASV_202  ASV_21  ASV_226  ASV_23  ASV_24  ASV_257  ASV_306  ASV_6  ASV_61 | S:*Clavulina_amethystina*  S:*Hygrocybe_sp*  O:Platygloeales  S:*Hyphodontia_sp*  S:*Cuphophyllus_virgineus*  S:*Ceratobasidium_sp*  S:Postia_sp  S:*Inocybe_assimilata*  S:*Inocybe_hydrocybiformis*  S:*Inocybe_arenicola*  S:*Saitozyma_podzolica*  O:Agaricales  S:*Tomentella_sp*  S:*Lactifluus_sp*  S:*Sebacina_sp* | Basidiomycota |
|  | | ASV_224 | F:Glomeraceae | Glomeromycota |
|  |  | ASV_156 | S:*Mortierella_elongata* | Mortierellomycota |
|  |  | ASV_174 | S:*Mortierella_alpina* |  |
|  |  | ASV_3 | S:*Mortierella_pseudozygospora* |  |
|  |  | ASV_33 | S:*Mortierella_sp* |  |
|  |  | ASV_39 | S:*Mortierella_zonata* |  |
|  |  | ASV_65 | S:*Mortierella_sp* |  |
| N | connectors | ASV_54 | S: *Mortierella_sp* | Mortierellomycota |
|  |  | ASV_4 | S:*Archaeorhizomyces_sp* | Ascomycota |
|  |  | ASV_8 | S:*Archaeorhizomyces_sp* |  |
|  |  | ASV_18 | P: Ascomycota |  |
|  |  | ASV_80 | S: *Leohumicola_minima* |  |
|  |  | ASV_105 | S: *Xeromyces_bisporus* |  |
|  |  | ASV_108 | C: Archaeorhizomycetes |  |
|  |  | ASV_112 | S:*Archaeorhizomyces_sp* |  |
|  |  | ASV_175 | S: *Cylindrium_syzygi*i |  |
|  |  | ASV_225 | S: *Trichoderma_lixii* |  |
